# Surgical treatment of chronic aortic dissection with liver dysfunction due to constrictive pericarditis caused by IgG4-related disease: a case report

**DOI:** 10.1093/ehjcr/ytae471

**Published:** 2024-09-02

**Authors:** Akie Shimada, Taira Yamamoto, Shizuyuki Dohi, Daisuke Endo, Minoru Tabata

**Affiliations:** Department of Cardiovascular Surgery, Juntendo Nerima Hospital, Takanodai 3-1-10, Nerima-Ku, Tokyo 177-8521, Japan; Department of Cardiovascular Surgery, Juntendo Nerima Hospital, Takanodai 3-1-10, Nerima-Ku, Tokyo 177-8521, Japan; Department of Cardiovascular Surgery, Juntendo Nerima Hospital, Takanodai 3-1-10, Nerima-Ku, Tokyo 177-8521, Japan; Department of Cardiovascular Surgery, Juntendo University, Hongo 2-1-1, Bunkyo-ku, Tokyo 113-8421, Japan; Department of Cardiovascular Surgery, Juntendo University, Hongo 2-1-1, Bunkyo-ku, Tokyo 113-8421, Japan

**Keywords:** Liver cirrhosis, Ascites, Chronic aortic dissection, Systolic pericarditis, IgG4-related diseases, Case report

## Abstract

**Background:**

Severe liver failure with ascites may be associated with cardiac disease and may be the primary manifestation of constrictive pericarditis or aortic dissection. We report a case of a patient with a chief complaint of ascites for whom close examination revealed that the liver injury was attributed to constrictive pericarditis and chronic aortic dissection, with immunoglobulin G4 (IgG4)-related disease (IgG4-RD) as the primary cause.

**Case summary:**

A 72-year-old man presented to the emergency department with scrotal oedema and ascites. Initially, the patient was hospitalized in the Department of Hepatology. However, computed tomography (CT) revealed aortic dissection (DeBakey type II), pericardial thickening, and impaired right ventricular dilatation. Therefore, we performed an ascending aortic replacement. IgG4 staining of the aortic wall revealed an IgG4/IgG-positive cell ratio of 35%. Pathological examination did not confirm the diagnosis of IgG4-related aortitis; however, the patient was diagnosed with IgG4-RD because of decreased blood IgG4 levels in response to steroid medication and the presence of heterogeneous thickened lesions in the pericardium. The patient took prednisolone 5 mg/day for 1 month post-operatively. His IgG4 level decreased but re-elevated above the baseline value after discontinuation of oral medication.

**Discussion:**

Liver cirrhosis was suspected given the ascites, although a CT scan on admission confirmed insufficiency of systemic circulation due to cardiac constrictive pericarditis with aortic dissection. Despite the complexity of various pathologies in this patient, collaborative efforts and effective communication within the medical team enabled successful aortic surgery, averting life-threatening complications.

**Learning points**

- Aortic dissection may manifest with a variety of symptoms, including pain, and can be concealed by other symptoms.
- Severe liver injury is an essential surgical risk in cardiovascular surgery and must be evaluated for severity and treated according to the underlying disease.
- IgG4-related disease may be suspected based on surgical pathology; IgG4 levels directly correlate with disease severity and a marked response to oral steroids, but serum IgG4 levels rise again when steroids are discontinued.

## Introduction

At the initial hospital presentation of patients with ascites, it is difficult to promptly diagnose the cause of liver injury. Painless aortic dissection, a rare cause of liver injury, has an incidence of 6% and is often Stanford type A with high mortality if untreated.^1,2^ Despite painless initial symptoms and ascites as the main finding, constrictive pericarditis and aortic dissection—which combined may indicate autoimmune disease—must be considered as differential diagnoses.^3,4^ Immunoglobulin G4-related disease (IgG4-RD) is a rare inflammatory disease that presents with immune abnormalities, high blood IgG4 levels, and marked infiltration and fibrosis of lymphocytes and IgG4-positive plasma cells, with simultaneous or atypical swelling, nodules, and hypertrophic lesions in various organs. In the cardiovascular field, the disease presents with fibrosis and lymphocytic infiltration of the aorta and pericardium without calcification.^5^

We report a sporadic case wherein constrictive pericarditis and chronic aortic dissection caused liver damage. Further investigations revealed IgG4-RD as the primary disease.

## Summary figure

**Figure:**
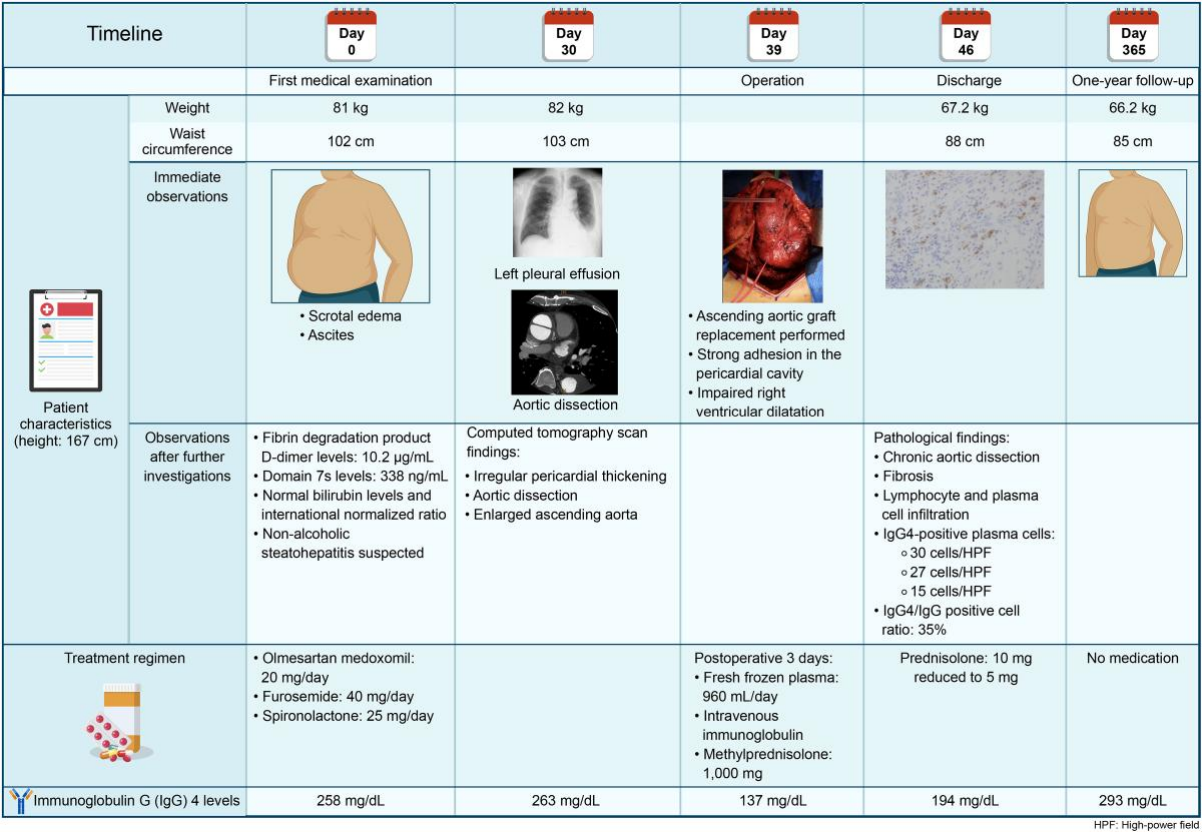


## Case presentation

A 72-year-old man, diagnosed with hypertension at 50 years of age without a history of hospital outpatient visits, presented to the emergency department complaining of scrotal oedema and abdominal distention. He had no history of surgery, allergies, or alcohol consumption, and no notable family history. He smoked 20 cigarettes/day. He was not taking any oral antihypertensives. Physical examinations revealed the following: blood pressure, 140/96 mmHg; height, 167 cm; weight, 81 kg; and waist circumference, 102 cm. His lower extremities were markedly oedematous. The patient was treated for ascites and hypertension with diuretics and hypotensive drugs, and laboratory tests for liver damage were initiated. Oral medication for 2 weeks was ineffective without ascites improvement. Computed tomography (CT) on the same day revealed a type II aortic dissection; the patient was admitted to the hospital.

Laboratory results indicated elevated cerebral natriuretic peptide and fibrin-fibrinogen degradation products, negative viral infection tests, and elevated type IV collagen 7s domain level (338 ng/m). His Child–Pugh grade A was 6. Non-alcoholic steatohepatitis was suspected as the cause of cirrhosis, and a liver biopsy was scheduled.

**Figure 1:**
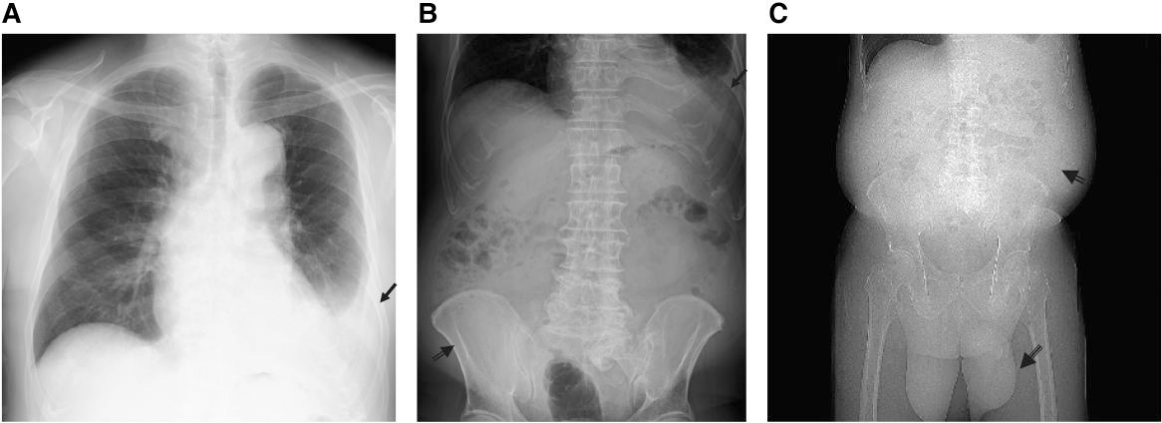
Pre-operative radiograph. (*A*) The single arrow indicates left pleural effusion. (*B*) The black double arrow indicates distended abdominal contents due to ascites. (*C*) The double arrows indicate the abdominal distended contents due to ascites, and the black triple arrow indicates the enlarged scrotum.

**Figure 2:**
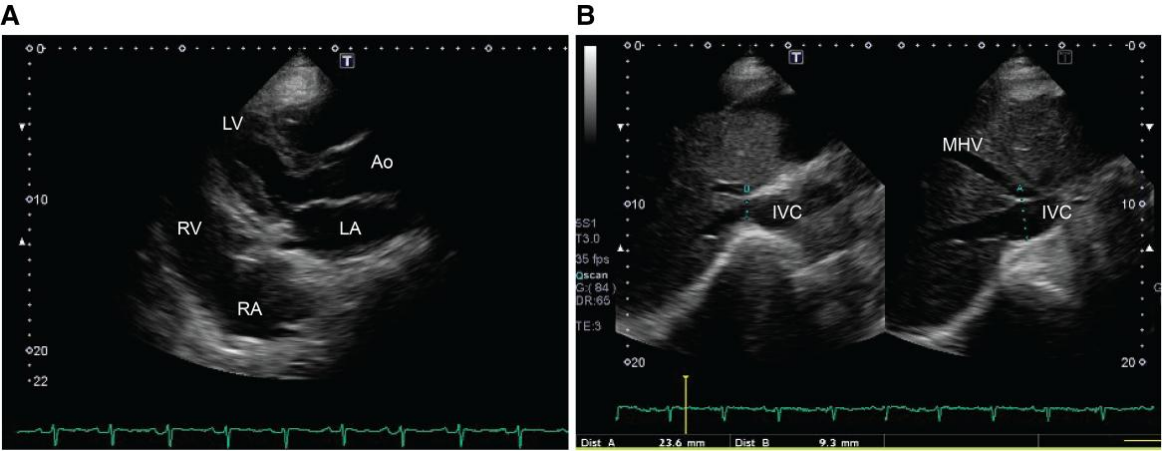
Pre-operative echocardiographic findings. (*A*) Parasternal long axis view reveals the left and right chambers with limited dilation and thickening of the right ventricular epicardium (white arrow). LV, left ventricle; LA, left atrium; RV, right ventricle; Ao, ascending aorta. (*B*) The IVC is dilated to 23.6 mm, with preserved respiratory variability. IVC, inferior vena cava; MHV, middle hepatic vein.

**Figure 3:**
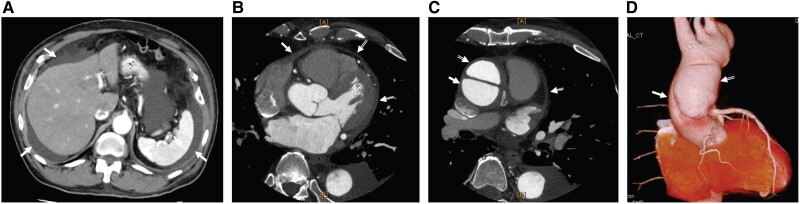
Pre-operative CT. (*A*) The arrows indicate ascites. (*B*) The pericardium around the ventricles is thickened, but the degree of thickening differs in each area: single, double, and triple arrows. No pericardial effusion is observed, and both the left and right ventricles are smaller than the left and right atria. RA, right atrium; LA, left atrium; RV, right ventricle; LV, left ventricle; V, sinus of Valsalva. (*C*) The ascending aorta is dissected; the double arrows indicate the false lumen, and the single arrow indicates the true lumen. The triple arrows indicate thickened pericardium. (*D*) Three-dimensional CT also shows a false lumen, indicated by double arrows, diagnosed as DeBakey type II aortic dissection.

Electrocardiography revealed normal sinus rhythm. Chest radiograph revealed a cardiothoracic ratio of 52% and left pleural effusion (*Figure 1*). Post-admission echocardiography revealed a left ventricular ejection fraction of 53% with no dysfunction and no valvular dysfunction at the left ventricular side. The estimated right ventricular pressure was 24 mmHg; nonetheless, there was moderate tricuspid regurgitation, and the inferior vena cava (IVC) was dilated (24 mm). The resting respiratory variability of the IVC was reduced to 22%, and the right ventricular acceleration time was shortened to 110 ms. Right ventricular dilatation restriction was suspected (*Figure 2A* and *B*). Abdominal contrast-enhanced CT revealed marked ascites and hepatomegaly (*Figure 3A*). A simultaneous chest CT revealed an irregularly thickened pericardial pericardium when the mediastinum was confirmed (*Figure 3B*). Moreover, a DeBakey type II aortic dissection with a false communicating lumen was identified. The ascending aorta was prominently enlarged (48 mm diameter), whereas the descending aorta was slightly enlarged (30 mm diameter) (*Figure 3C* and *D*).

We determined that ascending aortic replacement was necessary for the aortic dissection. The patient was considered to have cirrhosis. Subsequently, pre-operative risk assessment revealed a Model for End-stage Liver Disease (MELD)/Na score of 12, with an estimated 3-month mortality of 6%.

**Figure 4:**
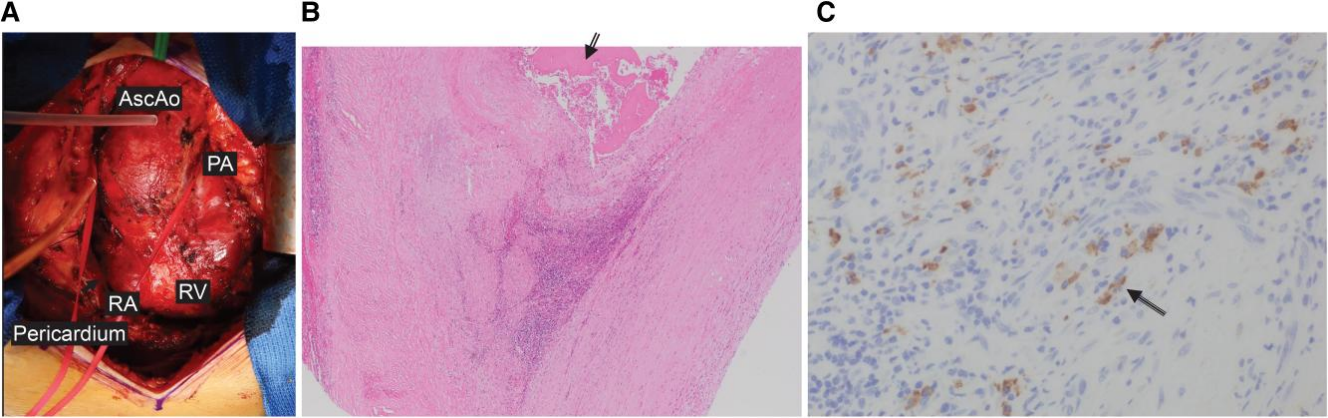
Intraoperative and pathological findings. (*A*) Pericardiotomy followed by adhesion debridement. The single arrows indicate the pericardium. All the adhesions are within the pericardial sac and are firm with high fibrosis. Red tape is placed around the ascending aorta. RA, right atrium; RV, right ventricle; PA, pulmonary artery; AscAo, ascending aorta. (*B*) Haematoxylin and eosin stain, ×100. Pathological findings of the ascending aorta; the double arrows indicate the dissected lumen with internal fibrin deposition. Cholesterin crystals are observed, and atherosclerosis is present, which are findings of chronic aortic dissection. Black arrows indicate dissection of the tunica media of the aortic wall. (*C*) IgG4 staining of the aortic wall. The triple arrows indicate IgG4 cells. The number of IgG4-positive plasma cells observed in three locations is 30 cells/high power field (HPF), 27 cells/HPF, and 15 cells/HPF, with an IgG4/IgG positive cell ratio of 35%.

On opening the pericardium, strong adhesions were observed (*Figure 4A*). Adhesions from the aortic root to the right atrium and anterior surface of the right ventricle were firm, and right ventricular dilatation was impaired. The pericardium exhibited fibrous thickening and no calcification. The adhesions were first resected. We performed an ascending aorta replacement with selective retrograde cerebral perfusion under hypothermia.

Intravenous methylprednisolone was administered intraoperatively and on post-operative Day 1. On post-operative Day 2, oral administration of prednisolone (10 mg/day) was resumed (*Figures 4A* and *B*). Tolvaptan 7.5 mg/day was also used to manage ascites and pleural effusion. Post-operatively, no complications were noted; the pleural effusion, ascites, and IgG4 levels had decreased, and the steroid dose was reduced to 5 mg/day. The patient was discharged 17 days post-operatively; his weight and abdominal circumference had reduced to 67.2 and 90 cm, respectively.

We suspected aortitis based on intraoperative pericardial thickening and adhesions and the nature of the aortic wall; additionally, post-operative blood samples were taken to confirm high IgG4 levels. Pathological examination revealed chronic aortic dissection. Despite no evidence of phlebitis obliterans in the aortic wall, fibrosis, lymphocyte infiltration, and plasma cells were observed. IgG4 staining of the aortic wall revealed the following IgG4-positive plasma cell counts in three locations: 30 cells/high power field (HPF), 27 cells/HPF, and 15 cells/HPF, with an IgG4/IgG-positive cell ratio of 35%.

Although IgG4-related arteritis could not be definitively diagnosed from pathological findings, we diagnosed the patient with IgG4-RD and continued the steroid therapy because of the elevated IgG4 cell count, decreased IgG4 levels in response to steroids on blood samples, and presence of extensive and heterogeneous thickened lesions in the cardiac pericardium. Six months post-operatively, the patient no longer required diuretics and only took prednisolone 5 mg/day; his IgG4 level decreased to 137 mg/dL. The patient discontinued the medication voluntarily and remained in good health 1 year post-operatively. CT 1 year post-operatively showed lymph nodes in the mediastinum, all < 5 mm in size, and bilateral common iliac arteries dilated to 22 mm. There were otherwise no other abnormal findings. However, subsequently, his IgG4 level increased to 293 mg/dL, prompting regular CT monitoring every 6 months.

## Discussion

The clinical problems in this case included the development of liver damage with ascites, an incidentally discovered painless aortic dissection, and the association of aortic dissection with cardiac constrictive pericarditis.

Aortic dissection can cause multiple organ failure manifestations, including cardiac tamponade and insufficient hepatic blood flow.^6^ Additionally, in the chronic phase, bloody pericardial effusion can cause constrictive pericarditis. Therefore, an unbiased diagnosis considering all bodily functions is essential to avoid diagnosis delays and impaired treatment.^2^

Hepatic dysfunction is a main contributor to poor post-operative prognosis in patients with aortic dissection. The MELD-XI score predicts liver injury risk.^7^ Pre-operative pericardial effusions can poorly impact the circulatory system and systemic haemodynamics, easily causing cardiac tamponade and poor perfusion of multiple organs.^8,9^ Lin *et al*. defined a MELD-XI score cut-off value for liver injury (MELD-XI ≥ 14) and reported that 60.9% of patients with aortic dissection developed liver injury.^8,10,11^

In this case, the aortic dissection was suspected to be related to constrictive pericarditis based on the intraoperative pathological findings; IgG4-RD was one of the differentials.^12,13^ The diagnostic criteria for IgG4-RD are fibrous masses in multiple organ systems and elevated serum IgG4 concentrations. Although this antibody plays little role in inflammation,^5^ in most affected patients, IgG4 levels directly correlate with disease severity.^13^ In IgG4-RDs, typically, 50% of the infiltrate comprises IgG4-producing plasma cells, and over 50 of these cells are visualized at high magnifications in three views.^12–14^ The characteristic IgG4-RD finding in this case was fibrosis with a relatively increased number of IgG4 cells; IgG4 levels recovered to normal in response to oral steroids.

Our findings are supported by previous studies.^5,12–14^ Furthermore, we identified the cause of the disease, which began with ascites, and treated it appropriately. A limitation of this report is that the patient had no history of hospital attendance before the aortic dissection; thus, precautionary measures could not be taken. Additionally, the condition of the aorta and pericardial sacs and IgG4 progression over time were unclear.

The diagnosis of patients with painless aortic dissection is challenging, and signs of right ventricular failure in the chronic phase may be fatal. Despite the hidden pathology in this case, life-threatening complications were averted with medical team cooperation and communication. Regular follow-ups are needed to prevent IgG4-RD recurrence.

## Patient’s perspective

This patient had no history of hospital visits due to an aversion towards hospitals but is grateful that this disease was detected and treated.

This patient was encouraged to submit a scientific article on his unique medical history.

## Lead author biography


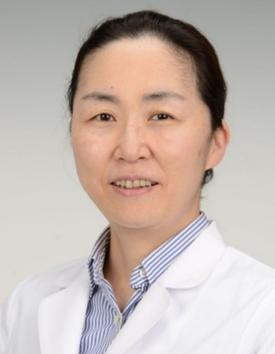


Dr Akie Shimada is a cardiovascular surgeon at Juntendo University Nerima Hospital, Tokyo, Japan. She received her MD in 2003 and her PhD in 2011 from Juntendo University School of Medicine (Tokyo, Japan). In 2018, she completed a cardiovascular surgery fellowship in Japan. Her research interests include valvular surgery and imaging evaluation of the aorta and aortic root pre-operative and post-operative.

## Data Availability

The data associated with this manuscript are not publicly available. However, relevant data and images are preserved in the hospital’s medical information system and can be made available by the corresponding author upon reasonable request.

## Notes

**Consent:** We obtained written consent from the patient in line with COPE guidelines to present this case report and disclose personal information.

**Funding:** Not applicable.
